# Harnessing Astrocytes and Müller Glial Cells in the Retina for Survival and Regeneration of Retinal Ganglion Cells

**DOI:** 10.3390/cells10061339

**Published:** 2021-05-28

**Authors:** Hyung-Suk Yoo, Ushananthini Shanmugalingam, Patrice D. Smith

**Affiliations:** Department of Neuroscience, Carleton University, Ottawa, ON K1S 5B6, Canada; hyungyoo@cmail.carleton.ca (H.-S.Y.); UshananthiniShanmuga@cmail.carleton.ca (U.S.)

**Keywords:** macroglia, astrocytes, Müller cells, optic nerve crush, retinal ganglion cells, spinal cord injury, signal transducer and activator of transcription 3, epidermal growth factor

## Abstract

Astrocytes have been associated with the failure of axon regeneration in the central nervous system (CNS), as it undergoes reactive gliosis in response to damages to the CNS and functions as a chemical and physical barrier to axon regeneration. However, beneficial roles of astrocytes have been extensively studied in the spinal cord over the years, and a growing body of evidence now suggests that inducing astrocytes to become more growth-supportive can promote axon regeneration after spinal cord injury (SCI). In retina, astrocytes and Müller cells are known to undergo reactive gliosis after damage to retina and/or optic nerve and are hypothesized to be either detrimental or beneficial to survival and axon regeneration of retinal ganglion cells (RGCs). Whether they can be induced to become more growth-supportive after retinal and optic nerve injury has yet to be determined. In this review, we pinpoint the potential molecular pathways involved in the induction of growth-supportive astrocytes in the spinal cord and suggest that stimulating the activation of these pathways in the retina could represent a new therapeutic approach to promoting survival and axon regeneration of RGCs in retinal degenerative diseases.

## 1. Introduction

The retina originates from the CNS during embryonic development [1]. The inner most layer of the retina harbors RGCs whose axons form the optic nerve that directly relays visual information to the brain [2]. RGCs can be considered CNS neurons because the optic nerve is myelinated by oligodendrocytes and do not regenerate spontaneously after injury [2,3]. Hence, the optic nerve crush (ONC) model has been widely used to determine the molecular mechanisms of neuronal survival and axon regeneration in the CNS [3,4,5,6,7,8,9]. While genetic and pharmacologic manipulations have been shown to activate neural repair mechanisms in RGCs after ONC, activation of macroglia in the retina, particularly astrocytes and Müller cells, has been shown to exert either detrimental or beneficial effects on survival and regeneration of RGCs after the injury [3,10,11,12,13,14,15].

Astrocytes in the CNS become activated in response to neuronal damage and neuroinflammation and form a dense network encapsulating the lesion site, known as the glial scar [15,16]. Although the glial scar functions as a physical and chemical barrier against further exposure to inflammatory agents, it also prevents growth of axons into the lesion site [16,17]. Astrocytes, therefore, have long been associated with the failure of axon regeneration in the CNS, and studies have attempted to eliminate or inhibit astrocytes to promote axon regeneration after CNS injury [16,18,19,20]. However, accumulating evidence now supports the concept that astrocytes are required for successful neuronal survival and axon regeneration in the CNS [12,13,16,19,21,22,23,24,25]. In this review, we will outline the evidence that reactive gliosis is required for successful neural repair in the CNS and suggest that harnessing the function of macroglia in the retina could promote survival and axon regeneration of RGCs.

## 2. Macroglia in the Retina

There are two types of macroglia in the retina: astrocytes and Müller cells. During retinal development, astrocytes from the brain enter the retina along the developing optic nerve [26,27]. In the mature retina, they are confined to the nerve fiber and ganglion cell layers [28]. On the contrary, Müller cells, the largest glial cell in the retina, originate from the retinal epithelium and span the entire retinal thickness [29,30]. The somata of Müller cells are located at the inner nuclear layer, and they extend their processes toward the outer and inner limiting membranes [31]. Müller cells ensheath retinal neurons and blood vessels in the plexiform and nerve fiber layers, allowing metabolic exchange between retinal vasculature and RGCs [32]. Astrocytes play a vital role in the development of the vascular system in the retina and contributes to the formation of the blood-retinal barrier [33,34]. Unlike Müller cells, astrocytes only envelop blood vessels in the nerve fiber and ganglion cell layers [32]. Astrocytes and Müller cells together maintain the integrity of the blood-retinal barrier by stabilizing tight junctions between endothelial cells and ensure the immune privilege of the eye [15,30]. They also provide essential nutrients, such as lactate and amino acids, from the circulation to neurons while participating in the retinal regulation of neurotransmitters, glucose metabolism and blood flow [10,15,30,35]. 

Astrocytes and Müller cells can provide RGCs with neurotrophic factors and antioxidants to maintain their viability [15,36,37,38]. Astrocytes can produce and release ciliary neurotrophic factor (CNTF), while Müller cells are known to be the major source of retinal brain derived neurotrophic factor (BDNF) and are capable of producing other well-known neurotrophic factors such as nerve growth factor (NGF) and glial cell line derived neurotrophic factor (GDNF) [14,39,40]. Increasing the retinal expression of these neurotrophic factors has been shown to promote survival and/or axon regeneration of RGCs. Moderate overexpression of BDNF in glaucomatous eye can result in long-term RGC survival while daily topical application of NGF can promote both survival and axon regeneration of RGCs after ONC [41,42]. Additionally, intravitreal co-administration of GDNF and CNTF can lead to survival and axon regeneration of RGCs after ONC possibly by directly binding to their respective receptors expressed by RGCs and/or inducing Müller cells to release additional neuroprotective factors, including BDNF and osteopontin [43]. Both macroglia can synthesize glutathione and counteract reactive oxygen species produced in the retina [11,15,36,37].

In response to the retinal injury, both astrocytes and Müller cells undergo reactive gliosis, up-regulating intermediate filaments, namely glial fibrillary acidic protein (GFAP), vimentin and nestin, and becoming more rigid [35]. This increased rigidness of both macroglia is mediated by signal transducer and activator of transcription 3 (STAT3), which is known as the master regulator of glial scar formation [18,35]. The rigidness allows for the glial scar formation, establishing the physical and chemical barrier to RGC axon regeneration [35]. Despite their proposed roles in inhibiting axon regeneration, studies show that these macroglia are rather essential in axon regeneration of RGCs. Astrocytes are known to release CNTF after lens injury and transform mature RGCs into a regenerative state, and the up-regulation of CNTF in astrocytes is also mediated by STAT3 [14,25]. Müller cells are also known to express CNTF after lens injury and hence may be involved in promoting the regeneration of RGCs in cooperation with astrocytes [44]. 

Since lens injury leads to intraocular inflammation, activation of inflammatory responses has been proposed to contribute to RGC axon regeneration [3,45]. Indeed, injecting the yeast wall extract zymosan can reproduce the regenerative effects of lens injury [3,14,44,45]. However, bacterial membrane component lipopolysaccharide (LPS) could not yield the same RGC axon regeneration, and this is due to zymosan’s unique ability to stimulate dectin-1 receptors on leukocytes that invade the eye in response to the intraocular inflammation [46]. This finding suggests that infiltrating immune cells may secret neurotrophic factors that ultimately stimulate RGCs to regenerate after ONC [46]. Indeed, both macroglia and macrophages have been proposed to be the source of neurotrophic factors that promote RGC axon regeneration [44]. However, studies have shown that depletion of macrophages from the eye does not reduce the regenerative effects of lens injury whereas reduced number of reactive macroglia compromised the beneficial effects of zymosan [44,47]. This suggests that macroglia may be the major mediators of the regenerative effects in response to the intraocular inflammation [44]. Considering the dual role of macroglia in RGC axon regeneration, they may exist in two reactive states: neurotoxic state and growth-supportive state. Since RGC axon regeneration could be induced specifically by lens injury, the reactive state of macroglia may depend on the type of injury. 

## 3. Two Distinct Reactive States of Astrocytes in the CNS

**Figure 1:**
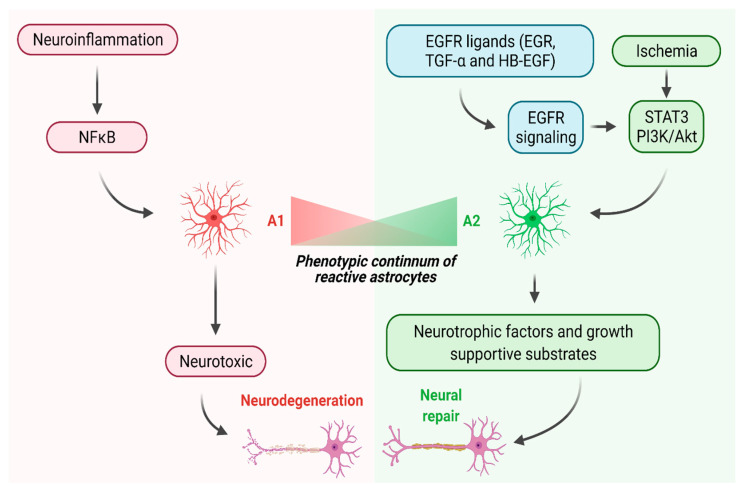
Neuroinflammation and ischemia lead to the generation of A1 and A2 astrocytes, respectively, in the spinal cord. Neuroinflammation may activate the NFκB pathway in reactive astrocytes and induce the A1 phenotype; on the other hand, ischemia may activate the STAT3 and/or PI3K/Akt pathways and induce the A2 phenotype. A1 and A2 astrocytes may exist on a phenotypic continuum. A1 astrocytes may be neurotoxic and promote neurodegeneration whereas A2 astrocytes may release neurotrophic factors and growth-supportive substrates and promote both survival and axon regeneration of CNS neurons. EGFR ligands may induce the generation of A2 astrocytes via activation of STAT3 and PI3K/Akt pathways (created with BioRender.com). Abbreviations: EGF, epidermal growth factor, EGFR, epidermal growth factor receptor, TGF-α, transforming growth factor-α, HB-EGF, heparin-binding EGF-like growth factor, NFκB, nuclear factor κB, STAT3, signal transducer and activator of transcription 3, PI3K/Akt, phosphoinositide 3-kinase/protein kinase B.

Pioneering studies by Sofroniew and colleagues established the concept that reactive astrocytes are necessary for protecting neurons from further damages after CNS injury and helping them survive and regenerate [17,20,23,48,49,50,51,52]. Additionally, accumulating evidence now posits the idea of phenotypical heterogeneity among reactive astrocytes [18,53,54,55,56]. Zamanian et al. have identified two distinct reactive states of astrocytes in the CNS using a transcriptome; they genetically profiled astrocytes after a systemic injection of LPS or cerebral ischemia [55]. LPS-induced neuroinflammation resulted in astrocytes expressing the components of classical complement cascade that are hypothesized to drive the loss of synapses and subsequently neurodegeneration [55]. On the contrary, the ischemic injury induced astrocytes to express neurotrophic factors and cytokines that can promote neural repair in the CNS [55]. The two types of reactive astrocytes induced by neuroinflammation and ischemia are known as A1 and A2, respectively [56]. Studies showed that the A1 phenotype is neurotoxic while the A2 phenotype is beneficial for neuronal survival and axon regeneration [56,57,58,59,60]. Figure 1 depicts the two distinct molecular pathways leading to changes in phenotype and function of reactive astrocytes.

It should be noted that ONC results in generation of A1 astrocytes [57]. Neutralizing the factors that induce the A1 phenotype, such as interleukin-1α, tumor necrosis factor α and complement component 1q, could prevent the A1 formation and RGC death up to 14 days after injury [57]. Currently, it is unknown whether lens injury can generate A2 astrocytes in the retina and whether this phenotypic division also exists among Müller cells. However, identifying growth factors and downstream effectors involved in the induction of the A2 phenotype would allow for developing therapeutic strategies for protecting RGCs and promoting axon regeneration. Considering that findings from the ONC model generally have been found to hold true for spinal cord injury (SCI) [3], molecular mechanisms of inducing the A2 phenotype in the spinal cord could be applied to the retina and may hold therapeutic potential for retinal degenerative diseases.

## 4. Harnessing Astrocytes to Promote Neural Repair in the Spinal Cord 

Astrogliosis has been extensively studied in the spinal cord, and accumulating evidence now suggests that it may have both beneficial and detrimental roles in the pathophysiology of SCI [18,23,51,52,56,61]. Although the signaling pathways leading to A1 and A2 phenotypes following SCI have yet to be determined, the nuclear factor κB (NFκB) and STAT3 pathways are believed to transform astrocytes into A1 and A2, respectively, because the roles of these two pathways seem to coincide with the hypothetical functions of A1 and A2 astrocytes (Figure 1) [61]. The NFκB pathway has a pivotal role in inducing neuroinflammation, and the inactivation of the pathway can reduce the expression of proinflammatory cytokines and significantly enhance the function of the spinal cord after SCI [62,63]. On the contrary, the inactivation of STAT3 pathway results in widespread infiltration of inflammatory cells and demyelination after SCI, whereas the activation of this pathway leads to rapid migration of reactive astrocytes to the lesion site to establish a physical barrier against inflammatory cells and promote significant improvement in functional recovery [64]. In further support of the idea that the STAT3 pathway may be involved in the generation of A2 astrocytes, Su et al. recently showed that down-regulation of microRNA-21 (miR-21) in astrocytes leads to STAT3-mediated conversion of A1 to A2 while up-regulation of miR-21 in astrocytes suppresses STAT3 activation and reverses the conversion process [60]. They also showed that these A2 astrocytes can promote axonal growth of neurons through the STAT3 pathway in vitro, suggesting that they may be beneficial to axon regeneration [60]. In addition to the STAT3 pathway, the phosphoinositide 3-kinase/protein kinase B (PI3K/Akt) pathway may also contribute to the generation of A2 astrocytes, as Xu et al. have shown that up-regulation of PI3K/Akt pathway and down-regulation of NFκB pathway are involved in counteracting A1 formation and promoting A2 formation [65]. Considering the evidence that the activation of STAT3 and PI3K/Akt pathways may be involved in the generation of A2 astrocytes, pharmacological stimulation of these pathways may lead to the increased population of A2 astrocytes that can promote neuronal survival and axon regeneration after CNS injury. The members of epidermal growth factor (EGF) family are known to activate these pathways via activation of epidermal growth factor receptors (EGFRs), and there is growing evidence that EGFR signaling can harness astrocytes to promote neuronal survival and axon regeneration in the CNS [21,22,66,67,68,69,70]. 

## 5. Manipulating Epidermal Growth Factor Signaling to Promote Neural Repair in the CNS

The EGF family is a group of related growth factors that are involved in a wide range of developmental processes, including proliferation, differentiation, and migration; the most notable members are EGF, transforming growth factor-α (TGF-α) and heparin-binding EGF-like growth factor (HB-EGF) [70]. These ligands signal through EGFR and three other homologous receptors, ErbB2, ErbB3 and ErbB4 [71]. Upon ligand binding, EGFRs undergo either homodimerization or heterodimerization; as an example, EGF can induce EGFR-EGFR homodimerization or EGFR-ErbB2 heterodimerization [70]. After this dimerization process, phosphorylated tyrosine residues function as docking sites for signaling protein complexes that are involved in PI3K/Akt and STAT3 pathways [70].

Members of the EGF and EGFR families are widely expressed in various regions of the CNS, including spinal cord, brainstem, cerebellum, diencephalon, telencephalon and hippocampus [70]. Their main function in the developing and adult CNS is to stimulate the proliferation and differentiation of neural progenitors; as an example, EGF and TGF-α stimulate both embryonic and adult striatal progenitors to proliferate and then differentiate into neurons and astrocytes [72,73,74]. The EGF and EGFR families also have been shown to be involved in neural repair after CNS injury. Studies showed not only that EGFR expression increases in subventricular zone (SVZ) after ischemic injury but also that intraventricular infusion of EGF promotes proliferation of neural stem cells in SVZ after cerebral ischemia and eventually leads to neuronal replacement in the injured striatum [75,76]. EGFR ligands can also exert neuroprotective effects against neurodegeneration; studies have shown that EGF and HB-EGF can promote dopaminergic neuronal survival in animal models of Parkinson’s disease [77,78]. 

Although EGF and EGFR have been shown to promote neurite outgrowth of cultured CNS neurons, retinal studies have shown that EGFR signaling is activated by growth-inhibitory molecules, and inhibition of the EGFR signaling can promote axon regeneration of RGCs [79,80,81]. Koprivica et al. showed that myelin-derived proteins Nogo-66 and oligodendrocyte myelin glycoprotein can trigger indirect phosphorylation of EGFR in cultured postnatal cerebellar granule cells by activating their common receptor complexes that consist of Nogo-66 receptor (NgR) and its co-receptors p75/TROY and Lingo-1 [81]. Blocking the NgR-induced phosphorylation of EGFR using irreversible EGFR inhibitor PD168393 promoted neurite growth in retinal explant and axon regeneration in the ONC model [81]. The authors suggested that the EGFR inhibitor acts directly on RGCs to block their growth-inhibitory responses to myelin-derived proteins [81]. However, Douglas et al. later showed that the EGFR inhibitor has no direct impact on RGCs and, surprisingly, EGFR [82]. They reported that EGFR is only activated in glial cells, such as astrocytes and oligodendrocytes, in the retina and optic nerve, 14 days after optic nerve injury [82]. Furthermore, siRNA-mediated knockdown of EGFR in retinal culture could not promote neurite growth, but addition of competitive EGFR inhibitor AG1478 could restore neurite growth to the siRNA-treated cultures, suggesting that EGFR itself does not mediate the inhibition of axon regeneration [82]. Although the authors could not pinpoint the exact target of the EGFR inhibitor, they provided in vitro evidence that the inhibitor stimulates the release of neurotrophins, such as BDNF and NGF, from RGCs and retinal glia, and increases cyclic adenosine monophosphate, a second messenger involved in axon regeneration [82].

Currently, it is unclear whether EGFR signaling is involved in inhibition of axon regeneration. However, in support of the possibility that EGFR signaling could support axon regeneration in the CNS, a couple of studies have shown that TGF-α can promote axon regeneration after SCI [21,22]. Based on previous evidence that astrocytes can promote neuroprotection and may support axonal growth after injury, White et al. hypothesized that endogenous astrocytes could be harnessed to support axon regeneration with proper stimulation after SCI, and they intrathecally administered TGF-α in adult mice for two weeks following the injury [21]. TGF-α was able to stimulate proliferation and migration of astrocytes toward the lesion center and promote axon regeneration within the lesion [21]. As EGFR immunoreactivity was the strongest in GFAP-positive cells, the authors suggested that TGF-α acts directly on astrocytes [21]. Interestingly, TGF-α treatment not only increased the expression of neurocan, which is associated with inhibition of axonal growth, but also increased the expression of laminin throughout the lesion site [21]. As laminin immunoreactivity was co-localized with axons, the authors suggested that astrocytes may contribute to the formation of basal lamina structures throughout the lesion and provide a permissive substrate for axon elongation [21]. White and colleagues later published another study showing not only that TGF-α treatment can transform astrocytes into a growth-supportive phenotype that supports robust neurite outgrowth of dorsal root ganglion cells in vitro but also that overexpression of TGF-α in vivo by intraparenchymal adeno-associated virus injection adjacent to the injury site increased axon regeneration at the rostral lesion border [22]. In support of these findings, Sofroniew and colleagues included EGF as a part of the combinatorial pharmacological treatment for CNS axon regeneration, showing that EGF can increase the release of axon growth-supportive substrates, including laminin, fibronectin and collagen, and contribute to the overall axon regeneration after SCI [68]. They also noted that although EGF significantly increased astrocyte proliferation and density, axons were able to grow through and beyond glial scar formation [68]. Recently, Chen et al. showed that EGF can generate A2 astrocytes in vitro by down-regulating A1-like genes and up-regulating A2-like genes, further supporting the previous findings [66]. Overall, it is evident that manipulation of EGFR signaling can aid CNS axon regeneration by inducing the A2 phenotype that can provide axon growth-supportive substrates.

## 6. Manipulating EGFR Signaling in the Retina

**Figure 2:**
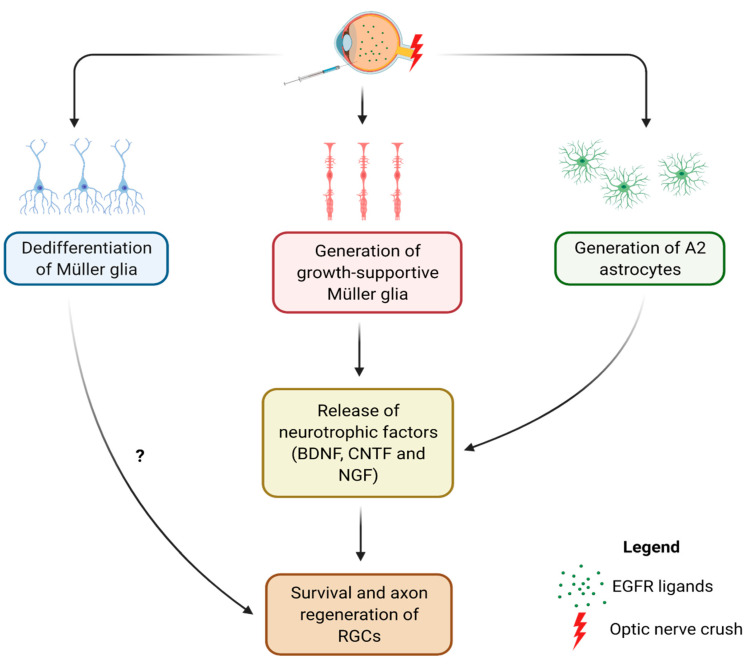
Intravitreal injection of EGFR ligands after ONC may lead to generation of A2 astrocytes and promote survival and axon regeneration of RGCs. It may also induce Müller cells to become more growth-supportive and help maintaining survival of RGCs and promoting optic nerve regeneration in cooperation with A2 astrocytes. Alternatively, Müller cells may dedifferentiate into retinal progenitor-like cells and contribute to survival and axon regeneration of RGCs (created with Biorender.com). Abbreviations: BDNF, brain derived neurotrophic factor, CNTF, ciliary neurotrophic factor, NGF, nerve growth factor, RGC, retinal ganglion cell, EGFR, epidermal growth factor receptor.

Although EGFR signaling seems to induce the A2 phenotype in the spinal cord, it is currently unknown whether EGFR signaling can also generate A2 astrocytes in the retina and promote RGC survival and axon regeneration after injury. Ever since the discovery of the intrinsic ability of mature RGCs to regenerate their axons, the major focus of ONC studies has been on further deciphering how to activate the intrinsic growth ability of RGCs [4,5,6,7,8,9]. Despite the evidence that retinal astrocytes and Müller cells can provide neurotrophic support to RGCs and maintain their viability, the post-injury reparative roles of these retinal macroglia still need to be elucidated. Additionally, no studies have shown whether EGFR ligand-induced EGFR signaling can lead to survival and axon regeneration of RGCs. Figure 2 depicts three molecular pathways that may be induced by manipulating EGFR signaling in the retina after ONC.

EGFR signaling is involved in proliferation of retinal progenitor cells and macroglia during retina development, and retinal EGFR expression has been shown to decrease as the retina matures and loses its mitogenic response to EGFR ligands [83]. However, retinal injury can increase EGFR expression in adult retina, suggesting that the retina becomes more responsive to EGFR ligands after injury [83]. Whether intravitreal injection of EGFR ligands after ONC can induce A2 formation and promote RGC survival and axon regeneration has yet to be investigated. However, as Harder et al. recently suggested that astrocytes may exert protective effects on RGCs through EGFR signaling [84], it may be possible that activation of EGFR signaling in astrocytes may at least promote RGC survival (Figure 2). Interestingly, they also showed that the up-regulation of complement C3 in astrocytes is mediated by EGFR signaling and responsible for protecting RGCs against high intraocular pressure [84]. Because C3 is a known marker of A1 astrocytes [57], this finding does not support the idea that A1 phenotype is neurotoxic and raises the question whether so-called neurotoxic astrocytes are indeed detrimental to neurons. However, this could also imply that astrocytes perhaps exist as a continuum, with a heterogeneous population of A1 and A2, as previously suggested by Liddelow and Barres (Figure 1) [56]. Therefore, it is possible that those neuro-supportive astrocytes with increased C3 expression could have exhibited a genetic profile that may lie somewhere in the middle of the phenotypic continuum. Overall, future research could consider investigating: (1) whether EGFR ligands can promote RGC survival and/or axon regeneration after injury via A2 formation, (2) whether downstream targets of EGFR signaling are involved in the potential neuroprotective and regenerative effects of EGFR ligands, and (3) the phenotypic ratio between retinal A1 and A2 astrocytes after injury and after post-injury treatment with EGFR ligands.

Since the retina also contains Müller cells that undergo reactive gliosis along with astrocytes, dissecting the reparative roles of retinal gliosis would require understanding the post-injury molecular changes in Müller cells. Unlike reactive astrocytes, the phenotypic dichotomy of reactive Müller cells has yet to be defined although studies seem to suggest that Müller cells may also exhibit a similar continuum of reactive states [35,85]. Because these two macroglia both express EGFR and respond to EGFR ligands [83,86,87], they may undergo similar molecular changes after injury (Figure 2). However, what makes Müller cells unique is that they can dedifferentiate into retinal progenitor-like cells and become pluripotent in response to EGFR signaling (Figure 2) [35,87]. Although how this dedifferentiation of Müller cells may contribute to the overall reactive gliosis in the retina remains unknown, studies have suggested that inducing Müller cells to become pluripotent may be a therapeutic strategy for retina regeneration in retinal degenerative diseases, such as age-related macular degeneration and glaucoma [35,87,88,89]. Future studies could try to determine the continuum of reactive states of Müller cells and investigate: (1) whether EGFR ligand treatments can harness Müller cells to become like A2 astrocytes and/or dedifferentiate into progenitor-like cells that may also contribute to survival and axon regeneration of RGCs, (2) the potential interaction between Müller cell gliosis and Müller cell-derived progenitor-like cells, and (3) how this interaction may affect retinal astrogliosis and the overall survival and axon regeneration of RGCs.

## 7. Conclusions

Despite having an overall negative connotation for over a decade, reactive gliosis is now being recognized as an essential contributor to CNS repair [20,23,68]. Although its positive contributions to neuronal survival and axon regeneration in the spinal cord has been established, its potential roles in promoting RGC survival and axon regeneration have yet to be determined. However, a growing body of evidence suggests that retinal macroglia may be essential in aiding post-injury survival and axon regeneration of RGCs [14,15,44,45,47,84]. Considering that EGFR signaling can contribute to axon regeneration in the spinal cord and activate the STAT3 pathway, which is critical to generation of A2 astrocytes, future research should investigate whether EGFR ligand-mediated EGFR signaling can induce A2 formation and promote survival and axon regeneration of RGCs after injury. It is also critical to understand whether EGFR signaling can induce Müller cells to become like A2 astrocytes, as they undergo reactive gliosis along with retinal astrocytes in response to injury. Additionally, there has been increasing attention to the possibility to deliver neurotrophic factors by eye drops, and clinical studies suggest that this could represent a safe and effective strategy for treating retinal degenerative diseases [42,90,91,92,93,94]. Hence, future research should evaluate the efficacy of EGFR ligand-based eye drops after ONC and investigate whether the topical delivery of EGFR ligands could generate growth-supportive phenotypes of retinal astrocytes and Müller cells. Harnessing retinal macroglia to promote survival and axon regeneration of RGCs would contribute to designing and improving therapeutic strategies for degenerative retinal diseases.
